# Level of male involvement and associated factors in family planning services utilization among married men in Debremarkos town, Northwest Ethiopia

**DOI:** 10.1186/s12914-014-0033-8

**Published:** 2014-12-02

**Authors:** Mihretie Kassa, Amanuel Alemu Abajobir, Molla Gedefaw

**Affiliations:** GAMBY College of Medical Sciences, Bahirdar, Ethiopia; The University of Queensland, School of Population Health, P.O. Box: 4006, Brisbane, Queensland Australia

**Keywords:** Male involvement, Family planning services, Ethiopia

## Abstract

**Background:**

Men’s participation is crucial to the success of family planning programs and women’s empowerment and associated with better outcomes in reproductive health such as contraceptive acceptance and continuation, and safer sexual behaviors. Limited choice and access to methods, attitudes of men towards family planning, perceived fear of side-effects, poor quality of available services, cultural or religious oppositions and gender-based barriers are some of the reasons for low utilization of family planning. Hence, this study assessed the level of male involvement in family planning services utilization and its associated factors in Debremarkos town, Northwest Ethiopia.

**Methods:**

A community-based cross-sectional study was conducted from October to November, 2013. Multi-stage sampling technique was used to select 524 eligible samples. Data were collected by using semi-structured questionnaires. Epi Info and SPSS were used to enter and analyze the data; univariate, bivariate and logistic regression analyses were performed to display the outputs.

**Results:**

Only 44 (8.4%) respondents were using or directly participating in the use of family planning services mainly male condoms. The reasons mentioned for the low participation were the desire to have more children, wife or partner refusal, fear of side effects, religious prohibition, lack of awareness about contraceptives and the thinking that it is the only issue for women. Opinion about family planning services, men approval and current use of family planning methods were associated with male involvement in the services utilization.

**Conclusions:**

In this study, the level of male involvement was low. Lack of information, inaccessibility to the services and the desire to have more children were found to be the reasons for low male involvement in family planning services utilization. Governmental and nongovernmental organizations, donors and relevant stakeholders should ensure availability, accessibility and sustained advocacy for use of family planning services. The family planning programs should incorporate the responsibility and role of males in the uptake of family planning services.

## Background

The involvement of men in reproductive health (RH) matters is important to achieving key millennium development goals (MDGs) including reduction of maternal mortality and the prevalence and impact of HIV/AIDS [1]. Family-planning (FP) programs have focused attention primarily on women to space and/or limit excessive child-bearing and to reduce maternal and infant mortality; accordingly, most of the services including research and information campaigns used to emphasis on women. This has reinforced the belief that FP is largely a woman’s business, the man playing a very peripheral role [2].

In Ethiopia, FP was initiated four decades ago; however, even after such a long period of time, the service has been amongst the lowest in Africa with 15% contraceptive prevalence rate (CPR) and 36% unmet need for FP [3]. Several factors are incriminated for the low coverage of FP services including the desire to have more children, lack of knowledge about contraceptive use and where to find contraceptives, health concerns, religious prohibition, husband opposition and low involvement of males. Male involvement in RH services utilization encompasses the way men accept and indicate support to their partners’ needs, choices and rights including using contraception and their own reproductive and sexual behavior to promote observance of human rights and the need to enforce equity. Consequently, it is particularly relevant in male-dominant cultures where men already have an all-encompassing involvement in decisions pertaining to family and society [4].

The Ethiopian population policy emphasizes the expansion of FP services through clinical and community-based interventions to attain CPR to 65% by 2015. It also involves encouraging a range of positive RH and social behavior by men to help ensure women’s and children’s wellbeing [5]. Despite this fact, interventions to involve men in reproductive issues have been low, and yet studies addressing the level of male involvement are scarce in the study setting. Therefore, this study was designed to assess the level of male involvement in FP services and its associated factors in Debremarkos town, Northwest Ethiopia.

## Methods

### Study design, setting and period

A community based cross-sectional study was conducted to assess the level of male involvement in FP services and its associated factors among married males (to explore their experience more) in Deberemarkos town, Northwest Ethiopia; the estimated population of Deberemarkos town was 86,786, of which 41,657 ((47.9%) males and from which about 64% were married)) and 45,129 (52.1%) were males and females respectively. About six in ten women, 26972 (59.7%), use FP services currently with an estimated total fertility rate (TFR) of 4.3%. Due to patriarchal dominance, males’ attitude and practice towards the importance and use of FP methods affect women’s decision - making in the study area. The study was conducted from October to November 2013.

### Source and sample population

All married males living in Debremarkos town were the source population whereas randomly selected married males living with their spouses in the selected *kebeles* (least administrative units) were the sample population.

### Sample size determination

Epi-Info version 3.1.1 statistical software was used to calculate the sample size by using single population proportion formula with the assumption of proportion (p) for male involvement in FP to be 65.5% (i.e., p = 0.655) from previous study [3], 95% CI with 5% tolerable error and design effect of 1.5 (i.e., a strategy of incorporating sampling weights and the design variables into the analysis to avoid errors in inference in complex sampling schemes like multistage sampling [6].

Therefore,

$$ \mathrm{n}={\mathrm{z}}^2\mathrm{p}\left(1-\mathrm{p}\right)/{w}^2={(1.96)}^20.65\left(1\hbox{--} 0.65\right)/0.0{5}^2=349 $$

where,

z = confidence interval (with 95% level of certainty)

*w* = margin of error (5%)

p = proportion (65.5%, p = 0.655)

Using the design effect of 1.5 (i.e., 349*1.5 = 524), the total sample size was 524 married males.

### Sampling procedures

**Figure 1:**
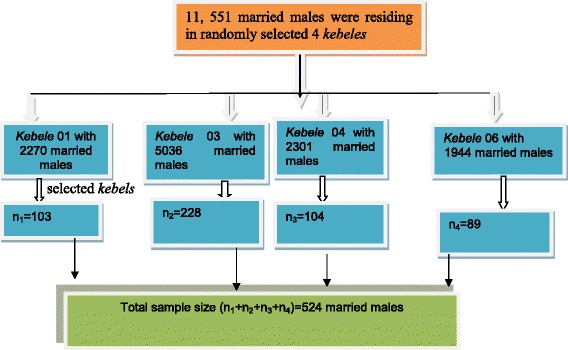
**Sampling procedure for level of male involvement in family planning among married men in Debremarkos town, Northwest Ethiopia, 2013.**

Multistage sampling method was used. Four of the seven *kebeles* of Debremarkos town were selected with replacement. A total of 20,183 households and 26,972 couples using FP methods currently were living in the town. By using random sampling method, a total of 524 married males were selected from 11,551 husbands residing in randomly selected 4 *kebeles*. A household would be the basic sampling unit in each *kebele* and samples were allocated proportionally to each based on their total household. Households were selected systematically by standing in the middle of the *kebeles* (as identified by the spinning pencil). Every household where the direction of the pencil point would be included in the study. If the boundary of the first *kebele* would be limited without getting the required number of respondents, the interviewers turned in the direction of their right hand and continue with the same sampling procedure until the required number was obtained (Figure 1).

### Data collection procedure

Data were collected using interviewer administered semi-structured questionnaires. The questionnaires were developed in English and translated into local language (*Amharic*). The questionnaire was designed to include socioeconomic variables, knowledge, attitudes and practice towards FP methods. In case where the respondent was not found after repeated visits, the immediate (nearby) household was interviewed. Two health officer research supervisors and ten health extension worker data collectors were trained and undertook the overall data collection activities under the immediate superviovion of the principal investigators for possible guidance.

### Data quality control

To assure data quality, the questionnaires were pretested (10% of the total sample) on similar population a week before the commencement of the main research. Two days training was given for data collectors and supervisors. Data were cleaned by using SPSS before analysis.

### Data processing and analysis

The data were handled confidentially and entered into SPSS version 16 statistical program for analysis. Frequency tables, graphs and proportions were used to present the data; logistic regression was used to test the associations.

### Variables of the study

#### Dependant variable

Male involvement in FP services.

#### Independent variables

Socio-economic and socio-demographic characteristics, approval of FP, spousal communication, knowledge, attitude and practice of contraceptive methods.

### Definitions

#### Male involvement

Based on the summative score of questions designed to assess male involvement in FP services, men with score 60% and above were considered as having better involvement in FP services.

### Positive attitude

Based on the statements assessing attitude, the mean score 3/5 (60%) of the distribution was considered as having positive attitude towards family planning [5].

### Knowledge

Based on the summative score of questions designed to assess knowledge, men with above the mean of the distribution or 60% were considered as having better knowledge of family planning services [5].

### Ethical consideration

Ethical clearance was obtained from the Research and Publication Directorate of Debremarkos University and supporting letter was obtained from Debremarkos town administration. The purposes of the study were explained and informed consent was obtained from all participants. Confidentiality and privacy were maintained throughout the study process by excluding identifications in the questionnaires.

## Results

### Socio-demographic characteristics

All currently married men in the study responded to the questionnaires making the response rate 100%. Mean age of the respondent was 36.70 (SD ± 5.87) years. More than 50% of the respondents were within the age range of 31–40 years. More than 93% of the study participants were *Amhara* by ethnicity (Table 1).

**Table 1:** **Socio-demographic and economic characteristics of currently married men in Debremarkos town, Northwest Ethiopia, 2013**

**Variables**	**Frequency (n = 524)**	**Percent**
**Age (years)**		
31-40	278	52.4
41-50	141	26.6
18-30	97	18.3
51-64	8	1.5
>64		
**Religion**		
Orthodox	458	86.3
Protestant	41	7.7
Muslim	19	3.6
Catholic	6	1.1
**Ethnicity**		92.3
Amhara	490	3.4
Oromo	18	1.5
Tigray	8	1.5
Others	8	
**Educational status**		
Tertiary education	211	39.7
Secondary education	104	19.6
Primary education	119	22.4
No formal education	90	16.9
**Occupation**		
Employee	216	43.1
Business man	177	36.5
Others	107	20.2

### Reproductive characteristics

The average number of living children per man was 2.7 with SD of 0.63 and the average desired number of children was 3.2 (SD ± 0.66). About four in ten (39.3%) men preferred 3–4 years for birth spacing (Table 2).

**Table 2:** **Reproductive characteristics of married men, Zone Debremarkos town, Northwest Ethiopia, 2013**

**Variables**	**Frequency**	**Percent**
**Current living children**		
**0**	19	3.5
**1-2**	157	28.9
**3-4**	317	58.3
**>/=5**	31	5.7
**Birth spacing (years)**		
**1-2**	163	30.0
**3-4**	214	39.3
**>4**	67	12.3
**Do not decided**	80	14.7
**Desired number of child**		
**0**	5	0.9
**1-2**	62	11.4
**3-4**	294	54.0
**>/=5**	163	30.0
**Currently use FP men**		
**No**	480	91.6
**Yes**	44	8.4
**Use of purpose**		
**Birth spacing**	403	75.5
**Limiting birth**	112	21.0
**Others**	9	1.7

### Knowledge of modern contraceptive methods

Most of the study respondents (99.2%) reported that they had ever heard about FP methods. About 64.9% of respondents listed 2–3 family planning methods while 30% of the respondents reported 1–2 years interval between two consecutive pregnancies. Generally, 91.6% of the respondents had knowledge about modern FP methods (Table 3).

**Table 3:** **Modern contraceptive methods knowledge of married men in Debremarkos town, Northwest Ethiopia, 2013**

**Variables**	**Frequency**	**Percent**
**Ever heard about FP**		
**Yes**	520	99.2
**No**	4	0.8
**List FP methods**		
**1**	120	22.9
**2**–**3**	353	67.4
**>/=4**	50	9.5
**None**	1	0.2
**List benefits of FP**		
**List one**	352	67.2
**benefit**	166	31.7
**List 2–3**	6	1.1
**None**	355	67.7
**Knowledge of sterilization**		
**Yes**	169	32.3
**No**		
**Sources of information**		
**Radio/TV**	253	48.3
**Health**	203	38.7
**professional**	40	7.6
**Poster**	22	4.2
**Partner**	6	1.1
**News paper**	496	94.7
**Know any FP methods**		
**Yes**	28	5.3
**No**		

### Approval and spousal communication

**Figure 2:**
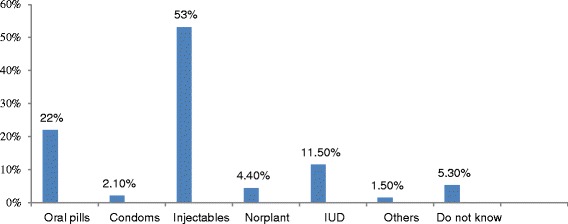
**Reasons not to use FP methods by married men in Debremarkos town, Northwest Ethiopia, 2013.**

More than half (54%) of married men discussed on such issues as when to achieve pregnancy, and/or prevent pregnancy and the use of contraceptives in the year prior to the study. On the other hand, 44.7% of men supported use of FP methods of their partners/wives. Almost one fifth (19.1%) of the respondents were neutral to approve use of contraceptives and 38.0% did not approve use of contraceptive while only 42.9% approved it. The reasons mentioned for the disapproval were the desire to have more children, wife or partner refusal, fear of side effects, religious prohibition, lack of awareness about contraceptives and the thinking that it is only the issue of women (Table 4, Figure 2).

**Table 4:** **Approval and spousal communication among currently married men in Debremarkos town, Northwest Ethiopia, 2013**

**Variables**	**Frequency (n = 524)**	**Percent**
**Discussion of FP(N = 524)**		
**Yes**	283	54.0
**No**	241	46.0
**Support use of FP**		
**Yes**	234	44.7
**No**	190	55.3
**Encourage use of FP**		
**Yes**	267	51.0
**No**	257	49.0
**Approved use of FP (n = 524)**		
**Yes**	225	42.9
**No**	199	38.0
**Neutral**	100	19.1

**Figure 3:**
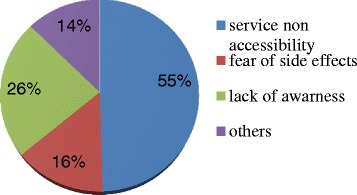
**Family planning methods used by respondent’s partners in Debremarkos town, Northwest Ethiopia, 2013.**

Almost all (99.4%) participants’ wives (spouses) were using contraceptive methods mainly injections (53.2%); however, 5.3% of the respondents did not know the methods used by their partners. Majority (75.5%) of the participants’ partners use contraceptive methods for child spacing while 21.0% use to limiting birth. On the other hand, only 8.4% respondents were using or directly participated in the use of FP methods mainly male condoms (Figure 3).

### Men’s attitude towards FP services

The attitudes of married men were assessed whether they had interest to know more about FP services; accordingly, 51.1% of the respondents had interest to know more about FP services but 48.9% of the respondents had no interest to do so and believed that it is a natural process. Men’s opinions about their roles in FP decision-making were assessed on a three-tier scale of agree, neutral and disagree. Accordingly, most male respondents disagreed that men should make decisions about selected FP issues in the family; 25% agreed that men should decide what to do when unwanted pregnancy occurs; 24.7% agreed that men should decide the types of FP methods; 49.8% agreed that FP practice services would make reduce confidence between husband; 31.6% agreed that men share the responsibility of FP services. Generally, 26.9% married men had positive attitudes towards the involvement of males in FP services utilization (Table 5).

**Table 5:** **Men’s attitude towards FP services among married men in Debremarkos town, Northwest Ethiopia, 2013**

**Variables**	**Frequency (n = 524)**	**Percent**
Interested to know about FP services		
Yes	268	51.1
No	256	48.9
Men should decide what to do when unwanted pregnancy occurs		
Agree	134	25.1
Disagree	282	52.8
Neutral	108	20.2
Men should decide the types of FP methods to use		
Agree	132	24.7
Disagree	289	54.1
Neutral	103	19.3
FP practice reduces confidence between husband and wife		
Agree	266	49.2
Disagree	142	26.7
Neutral	116	21.6
Men share the responsibility of FP		
Agree	169	31.6
Disagree	283	53.0
Neutral	72	13.4

### Logistic regression analysis

The binary logistic regression analysis of some independent variables in relation to the dependent variable was undertaken. Odds ratio (OR) with 95% confidence interval (CI) were used to assess the association between the variables. Men who had negative opinion about condom use with the believe that it reduces sexual potency were 2.13 times less likely to be involved in the use of FP services than those with positive opinion [AOR = 2.13, 95% CI: 1.28-3.53, p-value = 0.003]. Men who approved FP services utilization were 4.26 times more likely involved in FP services utilization than men who did not approve [AOR = 4.26, 95% CI: 2.51-7.22, p-value = 0.001]. Men who supported their wives to use FP methods were 1.61 times more likely involved in FP services utilization than men who did not support their wives to use FP services [AOR = 1.61, 95% CI: 1.10-2.35, p-value = 0.014]. Men encouraging their spouses to use FP were 1.74 times more likely involved in FP services utilization than men who did not encourage to do so [AOR = 1.74, 95% CI: 1.19-2.55, p-value = 0.005] and men who were using FP methods mainly condoms during the survey were 2.57 times more likely involved in FP services utilization than men who were not using condoms [AOR = 2.57, 95% CI: 1.30-5.01, p-value = 0.007] (Table 6).

**Table 6:** **Bivariate and multivariable analysis results of selected variables against male involvement in FP services utilization among married men in Debremarkos town, Northwest Ethiopia, 2013**

**Variables**	**Male involvement**		**COR**	**95% CI**	**AOR**	**95% CI**
	**Yes**	**No**				
Condom reduces sexual potency						
Yes	232	33	1.00	(1.21–3.10)**	1.00	(1.28–3.5)*
No	203	56	1.94		2.13	
Approve FP						
Yes (ref)	145	85	1.00	(2.66–7.37)***	1.00	(3–9.1)***
No	80	114	4.437	(1.09–3.06)*	4.257	(1.1–3.1)**
Neutral	29	71	1.825		1.817	
Support use of FP						
Yes (ref)	138	121	1.00	(1.42–3.3)***	1.00	(1.1–2.4)*
No	96	169	2.01		1.61	
Encourage use of FP						
Yes (ref)	124	135	1.00	(1.27–2.59)***	1.00	(1.19–2.55)***
No	89	176	1.82		1.74	
Currently using FP						
Yes (ref)	29	230	1.00	(1.01–4.02)*	1.00	1.29–507)*
No	15	250	2.101		2.57	

*p < 0.05 **p < 0.01; ***p < 0.001 (ref.) = Reference group.

## Discussion

Involving men and obtaining their support and commitment to FP services is crucial for increasing their uptake. This research assessed the level of men involvement in FP services utilization and highlighted the potential insights of men’s attitude towards FP services utilization in Debremarkos town, Northwest Ethiopia. The desire to have another child, lack of awareness, religious prohibition, fear of side effects, men’s attitudes towards FP use and others were among the reasons reported for low involvement of males in FP services utilization in the study area. Men’s knowledge on FP services was high as compared to previous study; this might be the result of interventions by the health sector and increased exposure to health education and media advocacy. The benefits and types of FP methods particularly for men were not well known by the study participants; however, the study documented positive association of men’s FP knowledge on couples’ contraceptive use and it complements with other studies [3,7]. The average living children per men and the desired number of children by the study participants were lower than study finding from Hosanna [8]; this could be explained by high knowledge of FP by the study participants. Family panning methods were used for birth spacing than limiting which is in agreement with other studies in Ethiopia and other developing countries [7,9].

In line with other studies, this study revealed negative attitude towards FP methods use among the respondents [7,10]. So, this negative attitude might affect the use of FP methods which is characterized by reduced contraceptive coverage. This indicated the need to introduce accurate information to develop positive attitudes towards the practice of the methods.

In this study the only modern contraceptive method used by males was condom; this finding was in line with the results from Tigray and Wolayita Soddo [3,7] but the attitude of married men towards condom utilization is negative [7,11]. Moreover, this study demonstrated that none of the respondents ever used sterilization. This might be partly due to the fact that none of the facilities in the study area provide male sterilization services to their clients and partly because the cultural norms against male sterilization [7]. Also some of the respondents had misconception about men permanent methods and did not agree on its effectiveness as well as acceptance as a method of choice showing direction to awareness creation sessions to motivate and reinforce others. Inter-spousal communication is an important intermediate step along the path to adoption and sustained use of FP services eventually [9,12]. Men’s report of the level of spousal communication about family planning and other reproductive health issues was quite poor in this study. A couple can come to a mutual decision on whether or not to use contraception to plan when to have children and how many to have through discussion [12]. In this study, we found low level of spousal discussion about the issue when compared with other studies [3,7,8]; the difference might be explained by cultural differences between the communities.

This study also revealed that married men’s support to use FP methods was less than half which is lower than other study findings [7,8]. Moreover, all men and women currently used short-acting methods for the purpose of child spacing rather than limiting. Similar results observed in other studies done in Ethiopia [2,3]. Thus, counseling of women in negotiating skills is necessary to develop confidence, influence their partner’s attitude towards fertility regulation to develop responsible reproductive and sexual behaviour among men [5]. Programs should also work and emphasize to reduce providers bias.

## Conclusions

Male involvement is not limited to the use of FP methods by itself rather to its supportive attitude that males have towards their spouses to usg FP methods and motivations in sharing responsibility in RH matters. In this study, the level of male involvement was low. Lack of information, inaccessibility to services and the desire to have more children were found to be some of the main reasons against male involvement in FP services utilization. Attitudes, spousal communications and approval were some of the factors associated with male involvement in FP services uptake.

Based on the study findings, the following measures were recommended:

- Governmental and nongovernmental organizations, donors and relevant stakeholders should ensure availability, accessibility and sustained advocacy for the use of FP services;
- The FP programs should incorporate the responsibility and role of males in the practice of FP service;
- Service delivering centers need to be properly equipped with materials to motivate males to use the services.
